# Home-based music therapy for persons with dementia and their spouses as primary caregivers

**DOI:** 10.3389/fpubh.2023.1250689

**Published:** 2023-10-03

**Authors:** Michal Rosenbach, Ayelet Dassa, Avi Gilboa

**Affiliations:** Department of Music, Bar-Ilan University, Ramat Gan, Israel

**Keywords:** dementia, primary caregiver, spouse, home-based, music therapy, musical strategies

## Abstract

Music therapy has been found to be an effective intervention for persons with dementia (PWD) and their primary caregivers (PC), yet the implementation of musical strategies to improve daily care in the home environment requires further exploration. This study developed and examined a home-based music therapy (HBMT) work model that offers weekly joint music therapy sessions, and additional bi-weekly phone-counseling sessions with the PC. This was followed by an additional 12-week support period that included 3 therapy sessions and 3 phone counseling sessions once every other fortnight, so that the same type of session occurred at a frequency of once a month. Participants were five couples (PWD + spouse as PC) who live in their home. Findings based on the qualitative multiple case study research method showed the importance of the music therapist’s (MT) continuous support. The MT’s presence made it possible to address the needs of both spouses, separately and together, while maintaining the required balance. Moreover, the MT’s presence enabled better implementation of the musical strategies independently and this was maintained during the intervention and the support period.

## Introduction

The number of persons with dementia (PWD) has increased parallel to the increase in life expectancy. Most live at home and are supported by a primary caregiver (PC) (1). Spouses who become the PC of a PWD cope with multiple challenges. The caregiver role they take on often becomes their main, and exclusive, identity, while their personal identity and needs are neglected (2). This adversely impacts the PC spouses’ quality of life, and they often feel emotionally overwhelmed, stressed, guilty, and burnt out (3).

Music therapy has been found to be a positive and beneficial intervention for both PWD and for their spouses as PCs. In recent years, various studies engaged in developing musical strategies for PCs have been conducted so that they can employ these strategies independently and daily without the ongoing presence of a music therapist (MT) (4). These strategies provide the PCs with musical tools that can help to reduce anxiety, depression, restlessness, confusion, and preserve existing capabilities of PWD (5). In most cases, guidance was provided in a limited number of sessions and the MT neither attended the caregiver-administered sessions nor provided further support. As a result, the spouses had difficulty implementing musical strategies over time, and more extensive support and counseling by the MT were necessary (6). In an international study, negative emotional reactions were also observed due to the lack of professional support of a MT during the intervention (7).

There is a large number of based-music interventions that are not conducted by a MT. However, studies in which the intervention included a MT’s presence indicate better implementation. A more extensive presence can be seen in Melhuish’s study (8) where a series of weekly music therapy sessions was conducted with spouses, with an emphasis on using music outside of the session as well. The results of the study showed that music helped to stimulate the PWD, improved mood, encouraged interaction, and strengthened the sense of connection and intimacy between the spouses. The MT’s presence, and therapy that was customized to the spouse’s needs, helped to better implement the musical strategies on a daily basis (8). A MT was also consistently present in another study (9) in which 12 weekly sessions were conducted with spouses at home, and concurrent phone counseling sessions were conducted with the PC every 2 weeks. The intervention was clearly positive and beneficial, but musical strategies were only used independently during the intervention period.

The purpose of the current study was to examine whether a work model that includes extensive support enables the independent implementation of music used in daily life and over time. Although the frequency of the support was gradually reduced to increase independence, we believe it is important to continue providing the couple with ongoing assistance.

## Methods

The current study employed principles of the qualitative multiple case study research method, which gathers a collection of specific cases and gleans insights using similarity or contrast (10). The study included five case studies so that they could be compared, conclusions reached, and the model examined from various aspects: from the MT’s, the PWD’s, and the PC spouse’s. Due to the need for brevity, the case studies will not be presented in full but the main themes that emerged from the data analysis will be presented.

This study was approved by the ethics committee of the Department of Music at Bar-Ilan University (E.MUS.2018-7), and the PCs signed their consent to participate in the study together with their spouses. Pseudonyms were used, identifying details were omitted, and the remaining information was kept confidential.

### Participants

The participants were recruited through organizations in Israel that provide home services to PWD. An explanation about the proposed program was given to the study participants. Criteria for participation included: (1) An individual with a diagnosis of dementia who lives at home with a spouse as a PC. (2) The spouse expressed their willingness to be involved in therapy and/or to receive phone counseling. (3) The spouse had difficulty coping with caring for the PWD at home. (4) The participants spoke Hebrew or English.

Five couples (a PWD + spouse as PC) participated in the study, ages 73–95. A diagnosis of dementia was given 3–7 years before the study began. Four spouses lived in assisted living and one couple lived at home. They were all assisted by a paid caregiver.

### Music therapy intervention

**Figure 1:**
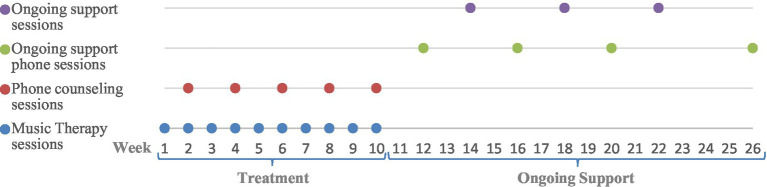
Schema of the study.

According to the model, the MT met with each couple at their home over 26 weeks, including a follow-up period, as follows: The intervention of 10 joint weekly music therapy sessions with both spouses, and five phone counseling sessions, which were held concurrently with the caregiving spouse. This was followed by an additional 12-week support period that included three therapy sessions and three phone counseling sessions once every other fortnight, so that the same type of session occurred at a frequency of once a month. Approximately 4 weeks after the support period ended, a follow-up session was held. Figure 1 presents the study’s schema with the therapy sessions and phone counseling sessions.

The intervention included singing, listening to music, dancing, or playing percussion instruments, according to the couple’s preferences. The musical strategies were personalized and tailored to each couple’s needs.

### Data collection tools

#### Recording the joint therapy sessions

All therapy sessions were recorded and transcribed. Transcriptions of the sessions included verbal and musical content and they enabled an inside look at what occurred in the intervention.

#### Recording the phone counseling sessions with the PC

All counseling sessions were recorded for documentation purposes. The talks were conducted in order to offer musical interventions and to examine whether these contributed to the daily care of the PWD. These sessions employed topics from the burden questionnaire (Zarit Burden Interview - ZBI) (11) for PCs to enable a discussion of common challenges with this population and to give legitimacy to address silenced topics, such as: “Do you feel anger/frustration when you are near your spouse?”; Do you feel that you have lost control of your life since your spouse’s disease?”

#### Follow-up phone call

Around a month after the intervention ended, a follow-up call was held with the spouse. This call examined whether the musical strategies had been implemented and how had the therapy impacted the daily life of the PWD and their spouse even after the intervention had ended.

#### Researcher’s journal

The journal included documentation of the treatment goals, preliminary planning of the therapy sessions, interpretation, feelings, and personal conclusions of the therapist-investigator that emerged during the intervention. Writing the journal led to an observation of the processes the therapist underwent with the clients and provided a necessary distance from the therapeutic process required as an investigator.

### Data analysis

The data was analyzed according to the cross-case synthesis method (12), which is standard with this method, and includes merging and crossing a collection of cases. Cross-case synthesis enables an in-depth exploration of similarities and differences across cases, to gain research insights. Data analysis was conducted following a three-stage process: (1) An in-depth analysis was conducted of each couple’s experiences during joint music therapy sessions, including preferred musical activities. (2) The case studies were compared to reveal similar or contrasting influences of the work model on the PWD, the PC, and both as a couple. (3) Identifying a repeating pattern made it possible to recognize central themes that emerged from all the case studies with the aim of drawing conclusions about the contribution of the work model.

## Findings

Five main themes emerged from an analysis of the content of all the couples’ sessions and phone counseling.

### Joint therapy strengthened the couple’s relationship

The study emphasized positive and joint musical work to facilitate moments of connection and quality time together for the couples. The joint therapy sessions contributed to strengthening the couple’s relationship, created a shared experience, and a break from the caregiver-care recipient roles that had been created as a result of the dementia.

*When Sophie and Mark sang the aria Habanera from Bizet’s opera Carmen together, it was easily possible to forget that Sophie had an advanced case of vascular dementia. In the therapy sessions the spouses sang songs together, Sophie as a soprano and Mark as a tenor-baritone. The restlessness and depression Sophie was coping with on a daily basis lessened and for the first time she was fit to participate in a joint activity with Mark. Mark had a huge smile on his face when Sophie joined him in song. Although it was without vocal warmup and sheet music, these were precious moments where he could put aside the heavy role of PC. They simply went back to being a couple, partners in a meaningful activity that gave them a sense of connection and enjoyment.*

### Practicing using music together helped with independent implementation

During the therapy sessions it was suggested that the couple practice using music together to help them on a daily basis. The joint practice made it possible to involve the PWD in therapy, fine-tune the intervention, and increase the PWD’s degree of cooperation.

*Jonathan’s walking was very slow and stiff since he was living with advanced Alzheimer’s and Parkinson’s, making it difficult for him to move from one place to another. However, when his wife Yael learned to use rhythm in the therapy sessions to encourage walking, it became easier, both technically and emotionally. Together we explored what was right for Jonathan: Whether to walk to the sounds of a marching tune, to the percussive sound of a regular and steady beat, or to a rhythmic count: left-right, or one-two. The practice was accompanied by laughter and enjoyment. Yael hummed a military march and humorously used military terms like: “Yes, sir” and saluted when Jonathan asked to go home for the weekend, and said “we are returning to base” when she guided him back to the couch, and “soldier at ease” when she helped him sit down. The practice became an experience, and Jonathan, whose speech was usually repetitive and meaningless, suddenly declared: “want a hug.” Jonathan’s involvement in the practice made him feel loved and equal and made the use of rhythm an inseparable part of the couple’s daily life.*

### The support sessions provided a separate therapeutic space for the PC

Concurrent to the joint therapy sessions, the PC spouse was individually addressed in a separate session, where topics that were inappropriate or impossible to discuss together could be raised and processed, giving this spouse an empathetic ear, understanding, and support. The support sessions made it possible for the spouse to share and ask for advice on the daily challenges common with dementia, to vent emotions, and to regain strength.

*Observing the spouse losing cognitive function and changing was intolerable for Tamar. Robbie was frustrated and depressed. Although music always played in their home, Tamar preferred to watch the musical activity rather than partake. The disconnection protected her. “I’ve lost a friend. I used to be able to consult and speak with him. Now I cannot,” Tamar shared for the first time, expressing the loneliness and pain she felt in living with her husband Robbie’s Alzheimer’s disease. We were already in the middle of the therapeutic process and Tamar felt safe to express these feelings out loud. She did not want to burden her children or close friends with these hard feelings. Outwardly, Tamar presented a strong and optimistic facade, kept busy, and was socially active. “But inside I’m a very sad person,” she expressed with honesty.*

*The validation, understanding, and empathy she received for her feelings during the phone sessions helped her to process and come to terms with the dramatic turning point in their lives. The therapy sessions created an opportunity for Tamar to return to the joint sessions and to experience positive and pleasant quality time with her spouse in a non-threatening manner. Also, the sense of distress Tamar described at the onset of the intervention lessened after she was given an opportunity to grieve the loss of their couplehood.*

### Connection between theoretical support and joint activity in therapy

The phone support sessions were held with the PC spouse separately. However, topics that emerged during the calls made it possible to fine-tune the intervention in the therapy sessions and to suggest different ways of using music. In this way, the phone sessions went from theory to applied practice.

*Singing and listening to classic Israeli songs turned out to be extremely meaningful for Eliraz. “There’s hope!” Motti, her husband, declared whenever she responded verbally and coherently after a musical activity. Motti was elated because Eliraz was living with dementia and spent most of the day sitting in her wheelchair, barely able to speak and understand instructions. However, when Motti tried to recreate the success on his own, he encountered great difficulty. He was frustrated that Eliraz did not respond to music on a daily basis in the same way she did during the joint therapy sessions. A joint examination revealed that without musical support, Motti had a hard time producing song and tended to recite the words without a melody. Also, he tended to ask Eliraz questions about the background of the songs, which elicited frustration and distress in Eliraz. Joint practice in the therapy sessions helped Motti learn singing techniques and how to address Eliraz in order to elicit past memories. Toward the end of the intervention, Motti succeeded in using music effectively. His singing was good, and he managed to create moments of closeness and connection, which the couple sorely lacked.*

### Ongoing support affected the use of music in daily life

When the therapy sessions ended, the participants continued to receive support for an additional 3 months. During this period, therapy sessions and phone support sessions were conducted with each spouse once every other fortnight, so that the same type of session occurred at a frequency of once a month. This frequency of sessions and calls made it possible for the couples to gradually wean off the support while still providing a small amount of assistance. In this way, the couples were encouraged to continue using music independently without being left to cope on their own.

*Elizabeth did not believe that a day would come when she and Victor would once again stand face to face, hold hands, and sway to the sounds of Tango. Victor, who was living with advanced dementia and required long-term care, was uncommunicative most of the time. The musical activities during the therapy sessions were a shared couple activity and elicited in Victor rare coherent verbal responses, which surprised and pleased Elizabeth. The phone support that Elizabeth received helped her to use musical strategies also during the week. Together we examined the couple’s challenges and how music can assist. Thus, Elizabeth learned to sing and play music in order to stimulate Victor, who spent most of the day sitting passively in the armchair. However, the musical work required effort from Elizabeth. She stated that the therapy sessions and the phone counseling boosted her motivation and encouraged her to use music more often. Without support, it was harder. In the follow-up phone session, about a month after the intervention ended, Elizabeth said: “After I speak to you, I also use singing, now I use it less.”*

## Discussion

The current study examined whether a work model that included extensive support for a total period of 26 weeks enabled the independent implementation of music, used in daily life and over time for PWD and their PCs. The work model made it possible to address the needs of both spouses, separately and together, while maintaining the required balance. Moreover, the MT’s presence enabled better implementation of the musical strategies independently and this was maintained during the intervention and the support. The research literature shows that addressing the roles of the MT for PWD and their spouses presents two widespread approaches. In the first approach, the MT focuses on treating the PWD but does not address the PC. The dependence on the therapist is high and the use of music is preserved only during the therapy sessions (13). In the second approach, the MT provides musical strategies as external counseling and not as part of a therapeutic intervention. There is no dependence on the MT, but the use of music is not maintained, and even negative effects are seen in the therapist’s absence (7). The current work model integrates both approaches, where the MT provides therapy as well as support in using musical strategies.

**Figure 2:**
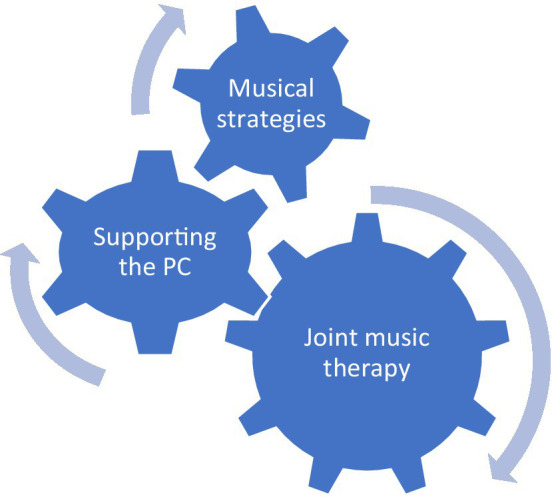
The MT’s roles in the work model.

As can be seen in Figure 2, the MT has three main roles: providing joint music therapy; supporting the PC spouse; and providing musical strategies for daily use.

The MT’s primary role is to provide therapy The therapy is given jointly while addressing the couple as one unit, contrary to focusing on the PWD and providing separate support for the PC (14). Strengthening the couple’s relationship when dementia is involved is a significant part of improving the couple’s quality of life. The PC spouses feel a sense of loss for their partner, since they can no longer share their thoughts, feelings, and experiences with them as a couple. The important sense of “us” and togetherness is damaged due to the disease (15).

The MT’s second role is to provide separate counseling and support for the PC. The phone counseling provided a platform for discussing topics that could not have been raised in the joint sessions, for giving tools to cope with the daily challenges, and for fine-tuning the therapeutic intervention. This model is consistent with other studies that see the importance and significance of addressing the PC spouse, in order to enable them to continue to provide optimal care (8, 16).

The MT’s third role is to enable the spouses to continue using music beyond the therapy session. In the current work model, musical strategies were given internally, as part of the joint therapy, which addressed the needs of both spouses. Musical strategies were also provided separately to the PC in a way that made it possible to fine-tune the therapeutic intervention and to practice during the session together with the MT. Unlike studies that include musical strategies conducted by the PC with limited guidance and support from the MT (17), in the current model the MT supports the couple in joint music therapy sessions and during a follow-up period. This format makes it possible to implement the musical strategies and provide ongoing support while gradually reducing the frequency of the sessions. The number of therapy sessions in the work model enabled an ongoing process to develop and sufficient time to practice and implement independent use of musical strategies. Only afterwards, it was possible to space out the therapy sessions and lessen the dependence on the MT.

## Conclusion

The study findings indicated that the MT’s extensive support helped to implement the use of music in the daily lives of PWD and their spouses. This extensive support requires an inclusive solution of couple’s therapy, counseling for the PC, and providing musical strategies. This inclusive solution requires extensive training of MTs. Alongside the resources required to enable the ongoing presence of a MT, it is a model that: (1) Provides a therapeutic space for the couples. (2) Enables good implementation of musical strategies. (3) Provides a stable foundation that makes it possible to reduce the frequency of the sessions, which saves resources, yet still provides support in using music independently. This study examined a limited number of couples and additional studies are required to further validate and develop the work model. In addition, a longer follow-up period is needed to examine the sustainability of the couple’s use of musical strategies and to explore whether music has been integrated into their daily lives.

## Data availability statement

The original contributions presented in the study are included in the article/supplementary material, further inquiries can be directed to the corresponding author.

## Ethics statement

The studies involving humans were approved by Department of Music at Bar-Ilan University (E.MUS.2018-7). The studies were conducted in accordance with the local legislation and institutional requirements. Written informed consent for participation in this study was provided by the participants’ legal guardians/next of kin. Written informed consent was obtained from the individual(s) for the publication of any potentially identifiable images or data included in this article.

## Author contributions

MR, AD, and AG conceived of the study, participated in its design, and took part in drafting the manuscript. MR, the music therapist, was in charge of coordination, data collection and coding as part of her Ph.D dissertation. AD and AG supervised the work. All authors contributed to the article and approved the submitted version.
